# Dose-dependent autophagic effect of titanium dioxide nanoparticles in human HaCaT cells at non-cytotoxic levels

**DOI:** 10.1186/s12951-016-0174-0

**Published:** 2016-03-22

**Authors:** Viviana R. Lopes, Vesa Loitto, Jean-Nicolas Audinot, Narges Bayat, Arno C. Gutleb, Susana Cristobal

**Affiliations:** Department of Clinical and Experimental Medicine, Cell Biology, Faculty of Medicine, Linköping University, 581 83 Linköping, Sweden; Department of Clinical and Experimental Medicine, Medical Microbiology, Faculty of Medicine, Linköping University, 581 83 Linköping, Sweden; Material Research & Technology Department (MRT), Luxembourg Institute of Science and Technology (LIST), 4422 Belvaux, Luxembourg; Department of Biochemistry and Biophysics, Stockholm University, 106 91 Stockholm, Sweden; Environmental Research and Innovation (ERIN) Department 41, Luxembourg Institute of Science and Technology (LIST), 4422 Belvaux, Luxembourg; IKERBASQUE, Basque Foundation for Science, 48013 Bilbao, Bizkaia Spain; Department of Physiology, Faculty of Medicine and Dentistry of University of Basque Country UPV/EHU, 48940 Leioa, Bizkaia Spain

**Keywords:** Autophagy, Cell-nanoparticle interactions, Dose, Keratinocytes, Titanium dioxide nanoparticles

## Abstract

**Background:**

Interactions between nanoparticles and cells are now the focus of a fast-growing area of research. Though many nanoparticles interact with cells without any acute toxic responses, metal oxide nanoparticles including those composed of titanium dioxide (TiO_2_-NPs) may disrupt the intracellular process of macroautophagy. Autophagy plays a key role in human health and disease, particularly in cancer and neurodegenerative diseases. We herein investigated the in vitro biological effects of TiO_2_-NPs (18 nm) on autophagy in human keratinocytes (HaCaT) cells at non-cytotoxic levels.

**Results:**

TiO_2_-NPs were characterized by transmission electron microscopy (TEM) and dynamic light scattering techniques. Cellular uptake, as evaluated by TEM and NanoSIMS revealed that NPs internalization led to the formation of autophagosomes. TiO_2_-NPs treatment did not reduce cell viability of HaCaT cells nor increased oxidative stress. Cellular autophagy was additionally evaluated by confocal microscopy using eGFP-LC3 keratinocytes, western blotting of autophagy marker LC3I/II, immunodetection of p62 and NBR1 proteins, and gene expression of LC3II, p62, NBR1, beclin1 and ATG5 by RT-qPCR. We also confirmed the formation and accumulation of autophagosomes in NPs treated cells with LC3-II upregulation. Based on the lack of degradation of p62 and NBR1 proteins, autophagosomes accumulation at a high dose (25.0 μg/ml) is due to blockage while a low dose (0.16 μg/ml) promoted autophagy. Cellular viability was not affected in either case.

**Conclusions:**

The uptake of TiO_2_-NPs led to a dose-dependent increase in autophagic effect under non-cytotoxic conditions. Our results suggest dose-dependent autophagic effect over time as a cellular response to TiO_2_-NPs. Most importantly, these findings suggest that simple toxicity data are not enough to understand the full impact of TiO_2_-NPs and their effects on cellular pathways or function.

## Background

Owing to its unique features, nanoparticles (NPs) can enter cells and interact with cellular machinery. Thus, cell-NPs interactions are been studied for their potential in nanomedicine [1–3]. Yet, current knowledge about the mechanisms underlying cell-NPs interactions is still limited. Though many NPs interact with cells without acute toxic responses, metal oxide NPs including titanium dioxide (TiO_2_)-NPs are known to induce dysfunction in autophagy associated with toxic effects [4–6]. Metal oxide NPs are more likely than others to have high autophagy properties due to their ability to increase oxidative stress and cationic damage [7, 8]. Thus, investigating the interactions of TiO_2_-NPs with biological systems, namely with autophagy is a fast-growing field in nanobiotechnology [7, 9].

Autophagy, referring to macroautophagy, is an evolutionarily conserved cellular quality control process that targets foreign and cellular components to the lysosomes for degradation [10]. Any cell growing in nutrient-rich conditions shows basal levels of autophagy that could be further induced upon stress. In the case of toxicant and/or metal-induced toxicity, autophagy may act as a survival mechanism. However, excess autophagy may also lead to cell death [11]. Therefore, tight regulation of autophagy is required; autophagy dysfunctions have been linked with numerous human pathologies including cancer, neurodegeneration, abnormal immune responses and premature ageing, so that autophagy regulation has become a new therapeutic strategy [12]. Autophagy dysfunction has been reported for diverse NPs such as liposomes, polymeric NPs, gold, iron oxide, zinc oxide, or titanium dioxide based NPs, carbon nanotubes, and quantum dots [9, 11].

The formation of the autophagosome, a double-membrane vesicle that surrounds the unwanted cellular elements, is the first step of the autophagy process. This vesicle is fused with lysosomes and subsequently targeted for degradation. A key protein in autophagosome formation is microtubule associated protein light-chain protein 3, LC3, which is converted from its cytosolic form (LC3 I) into an active membrane-bound form (LC3 II) by sequential proteolysis and lipidation during autophagosome assembly [10, 12]. Besides, other two proteins, p62/Sequestosome 1 (p62/SQSTM1) and NBR1 (Neighbor of BRCA1), play a role in cargo delivery to the autophagosome [13]. Both p62 and NBR1 are autophagic cargos that bind to LC3-II when conjugated with the autophagosome membrane [13, 14]. The link between NPs and autophagy can be evaluated from two perspectives, as potential autophagy disruptors and its impact in cell function; and how NPs-modulated autophagy could be explored for the development of therapeutic and biomedical applications [9, 15].

This study focuses on the autophagic effects of TiO_2_-NPs in human keratinocytes (HaCaT) cells. Since the discovery of NPs unique physicochemical properties, TiO_2_-NPs have been one of the most widely produced NPs, with at least 10,000 tons produced each year [16]. TiO_2_-NPs acceptance grew quickly due to their high stability, photocatalytic properties, and low toxicity of its counterparts, TiO_2_-fine particles. TiO_2_-NPs have been then incorporated in many consumer products from cosmetic, pharmaceutical, paints and food industries [15, 16].

Keratinocytes are a major component of the epidermis, the outmost skin layer and one of the first barriers interacting with NPs [17]. Whether or not TiO_2_-NPs penetrate damaged or unhealthy skin is dubious. Skin penetration studies have used both in vivo and in vitro models with intact cells or mechanically damaged skin, and various concentrations and treatment times [18, 19]. Most of the in vivo and in vitro studies for dermal treatment have concluded that TiO_2_-NPs do not penetrate the *stratum corneum* [19]. However, a study by Shi et al. provides evidence that TiO_2_-NPs (5–20 nm) can penetrate the skin and interact with the immune system [15]. In addition, the presence of 14 nm silica coated TiO_2_-NPs within the epidermis and superficial dermis has been observed [20].

Therefore, our goal was to use in vitro keratinocytes (HaCaT) to investigate the interactions of TiO_2_-NPs with cellular autophagy at non-cytotoxic doses. We used then uncoated TiO_2_-NPs (18 nm) to investigate the impact on cytotoxicity, ROS generation and uptake behavior under acute treatment to define the non-cytotoxic levels. Here we report that TiO_2_-NPs dose may shift the effects on autophagy from induction to blockage. These findings may open up the possibility of modulating autophagy by NPs through tuning their dose.

## Results

### NPs characterization

**Fig. 1:**
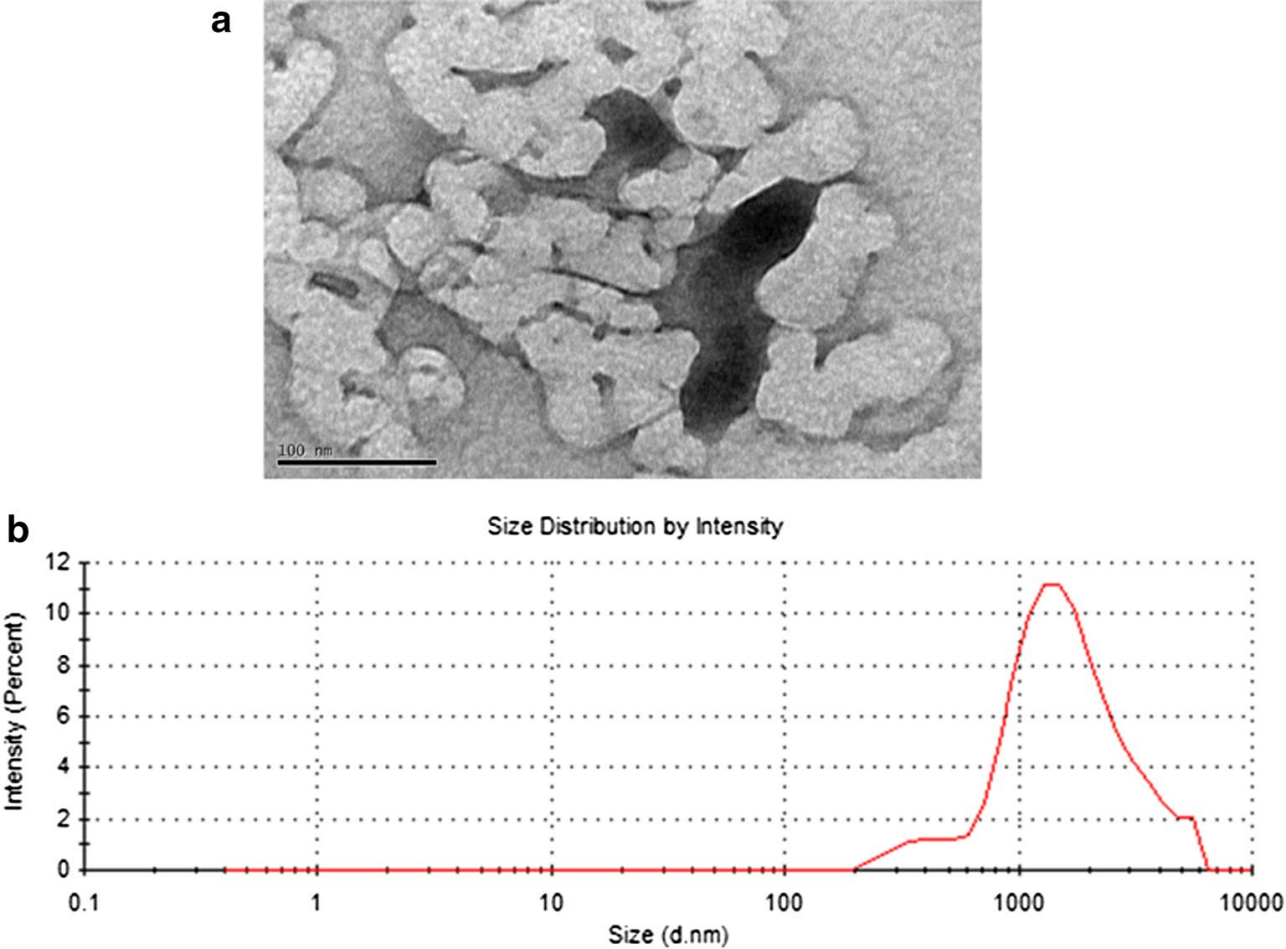
Characterization of TiO_2_-NPs in cell culture medium. **a** Representative TEM image of 18 nm TiO_2_-NPs in DMEM medium. **b** Dynamic light scattering analysis with TiO_2_-NPs suspended in DMEM cell culture medium. Analyses were performed from the stock solution (1 mg/mL). *Scale bar* (*bottom-left*) represents 100 µm

Characterization of TiO_2_-NPs was done by transmission electron microscopy (TEM), zeta potential (Z-potential) measurement and dynamic light scattering (DLS) in water and cell culture medium (Fig. 1 and Table 1). TEM images of TiO_2_-NPs exhibited a near-spherical shape and aggregates. The hydrodynamic sizes and zeta potentials of TiO_2_-NPs in both water and in cell culture media showed that TiO_2_-NPs suspensions were unstable and aggregating.

**Table 1:** Physicochemical properties of NPs

NPs	Purity %	Crystal structure	Size (nm)	Suspension	Hydrodynamic size (nm)	polydispersity index	Z-potential (mV)
TiO_2_	99+	Anastase	18	Water	1369.0 ± 27.97	0.307 ± 0.03	−5.59 ± 1.70
				DMEM	882.3 ± 29.54	0.300 ± 0.01	−8.55 ± 1.97

The characterization of NPs was performed in sterile deionized water with 10 mM NaCl and in cell culture medium (DMEM)

### TiO_2_-NPs are not cytotoxic and induce autophagosomes formation

**Fig. 2:**
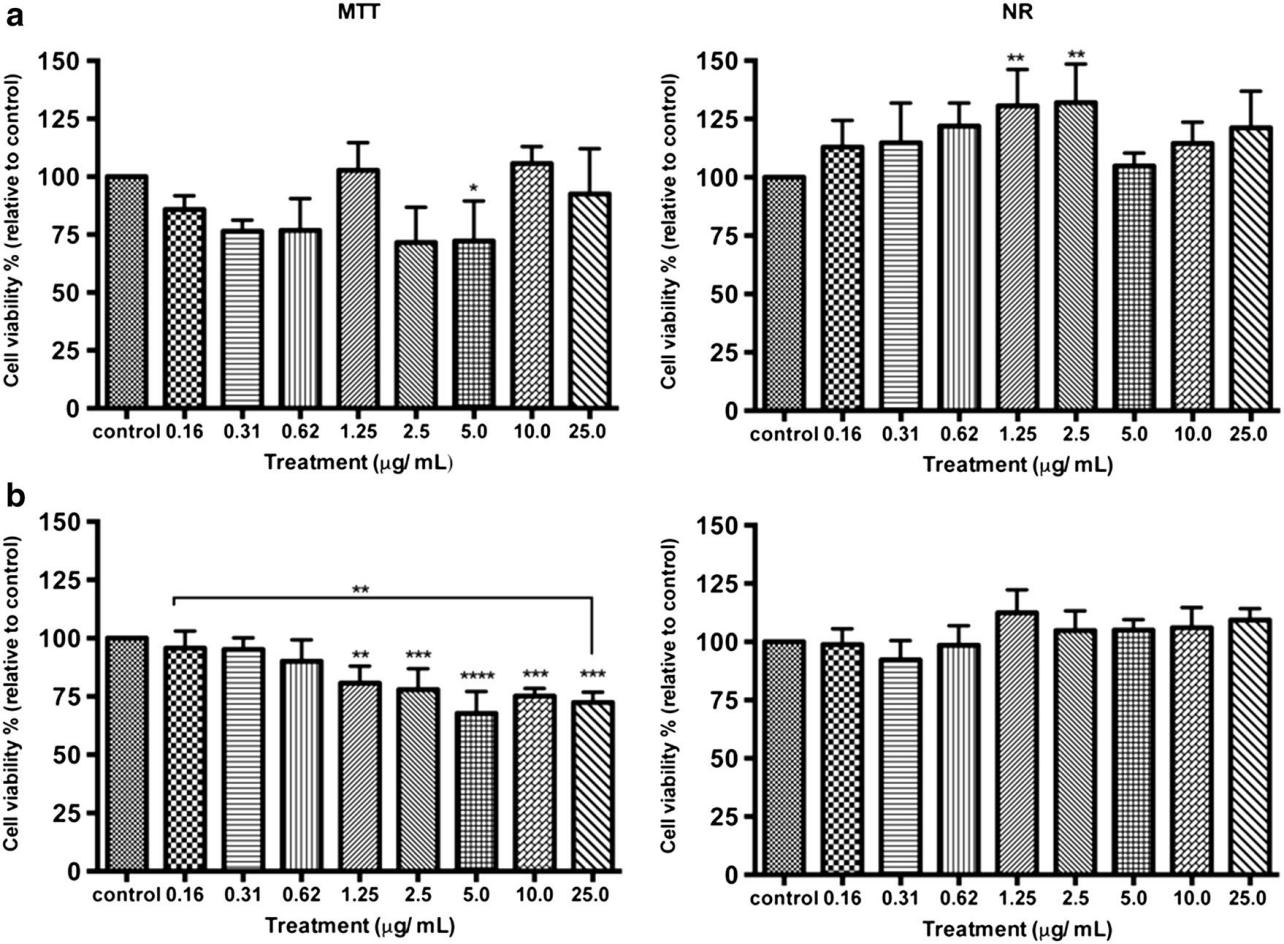
TiO_2_-NPs are not cytotoxic to HaCaT cells. Cells were treated to TiO_2_-NPs at doses ranging from 0.16 to 25 μg/mL. Cell viability was measured after 1 h (**a**) and 24 h (**b**) of treatment by MTT and NR assays. Data are presented as the mean % of cell viability relative to the negative control (cells without treatment). Values represent mean ± SD (n = 3). **P < 0.01, ***P < 0.001, ****P < 0.0001

To define a non-cytotoxic level of NPs on skin cells, the 3-(4, 5-dimethylthiazol-2-yl)-2, 5-diphenyl tetrazolium bromide (MTT) and neutral red (NR) assays were used after treating HaCaT cells with TiO_2_-NPs for 1 and 24 h at 0.16-25 µg/ml (Fig. 2a, b). Dose was selected based on modern sunscreens containing TiO_2_ between 2.5 and 9 % [16]. The MTT results show that TiO_2_-NPs induced a 15–25 % loss of cell viability still above the non-cytotoxic threshold of 70 % defined by the ISO standard [21]. The NR assay however reveals a slight increase of cell proliferation for both doses over time. These results however are not contradictory considering the principles of the assays. MTT assay is based on MTT conversion by mitochondrial enzymes whereas the NR assay assesses the neutral red dye uptake by functional lysosomes [22–24].

**Fig. 3:**
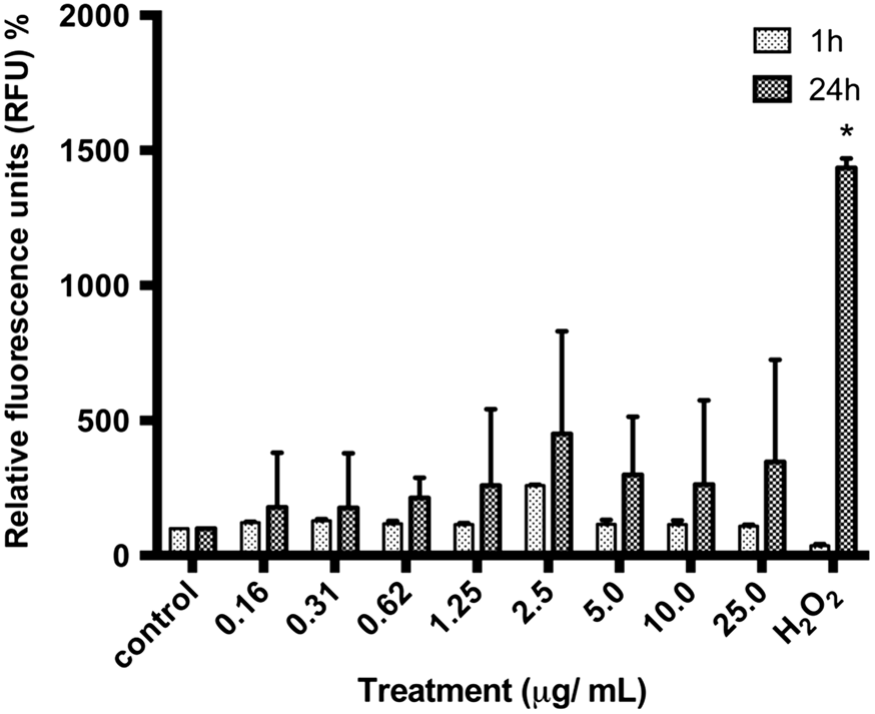
No production of oxidative stress by TiO_2_-NPs. Cells were treated to TiO_2_-NPs at doses ranging from 0.16 to 25 μg/mL during 1 and 24 h. The intracellular ROS was evaluated by DCFH-DA assay. Values represent mean ± SD (n = 3). No statistical differences in ROS generation were observed for any of the doses tested (*P <  0.05)

Overall, TiO_2_-NPs did not impair cell viability of skin cells after 1 or 24 h. We further evaluated reactive oxygen species (ROS) production induced by TiO_2_-NPs. We did not observe any increase of cellular ROS at 1 h nor at 24 h treatment (Fig. 3).

**Fig. 4:**
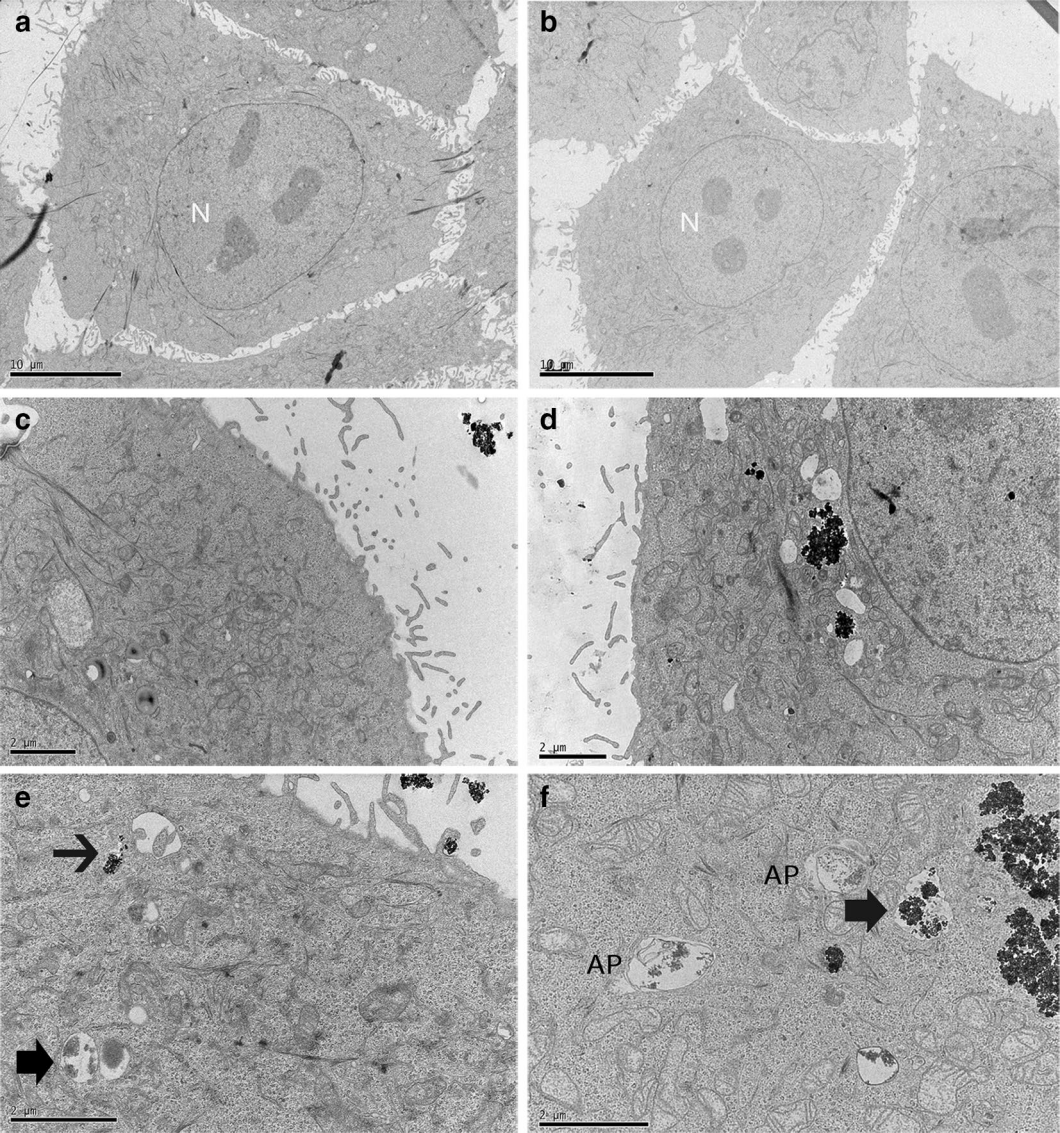
Intracellular uptake of TiO_2_-NPs by HaCaT cells and cellular distribution. TEM images of cells incubated at 37 °C and 5 % CO_2_ without and with TiO_2_-NPs at low-dose (0.16 μg/mL) and high-dose (25 μg/mL). **a** Control cells without NPs for 1 h; **b** Control cells without NPs for 24 h; **c** Cells exposed to 0.16 µg/mL TiO_2_-NPs for 1 h; **d** Cells exposed to 0.16 µg/mL TiO_2_-NPs for 24 h; **e** Cells exposed to 25.0 µg/mL TiO_2_-NPs for 1 h; and **f** Cells exposed to 25.0 µg/mL TiO_2_-NPs for 24 h. Morphological changes in cells treated. Intracellular accumulation of TiO_2_-NPs in autophagosomes like-structures (AP) and in vesicles (*short arrows*). Autophagic vesicle formation (*thin arrow*). *Scale bars* for **a**–**b** images are 10 µm and for **c**–**f** images are 2 µm. *N* nucleus

**Fig. 5:**
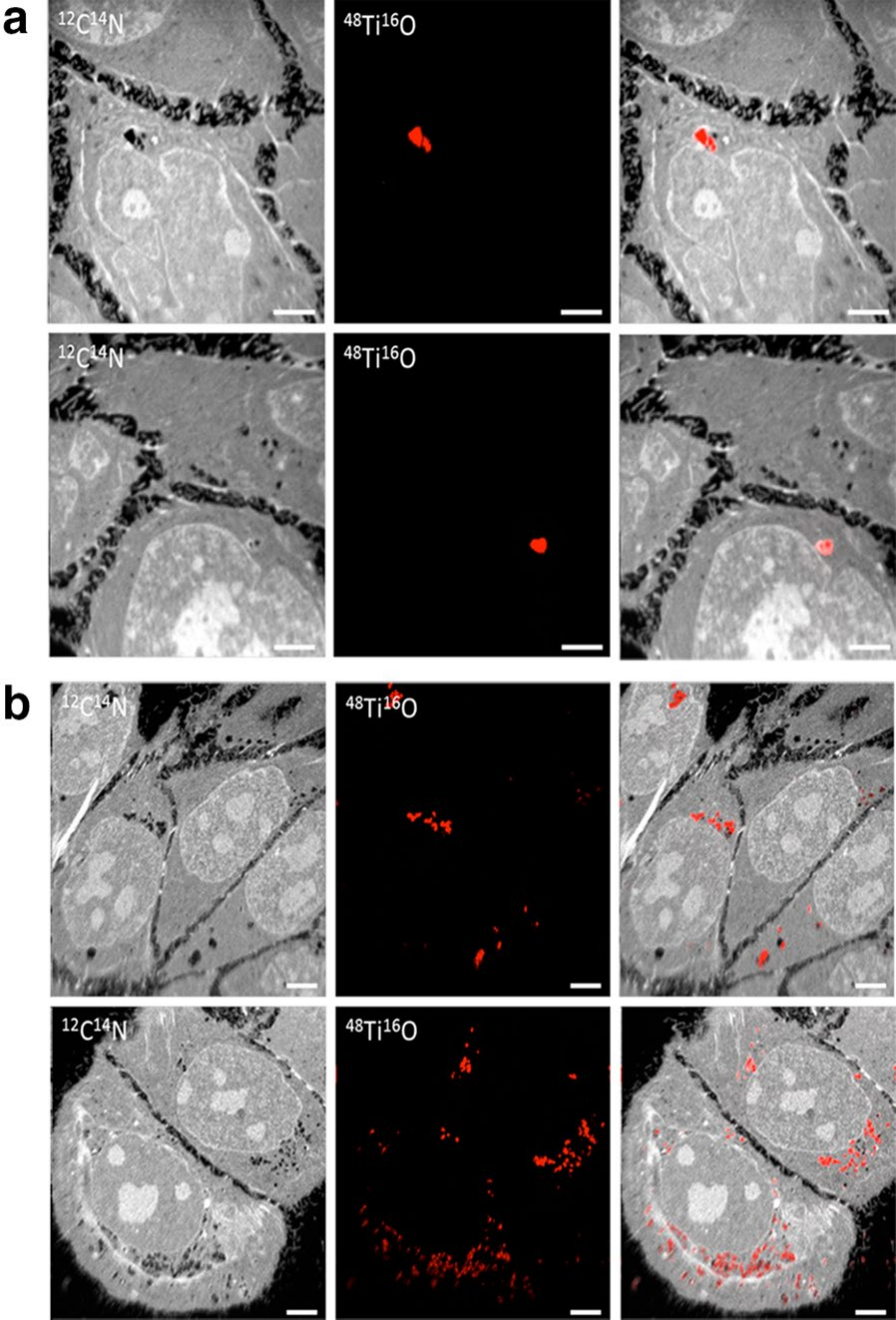
NanoSIMS confirms TiO_2_-NPs are uptake and are not inside nucleus. NanoSIMS images of the elemental distribution of 12C14N and 48Ti16O on an ultra-section cut of HaCaT cells exposed to TiO2-NPs at **a** low dose (0.16 µg/mL)—low dose and **b** high-dose (25.0 µg/mL)—high dose for 24 h. Acquisition time 30 ms/pixel. *Scale bars* represent 5 µm

The next step was to evaluate the cellular uptake and localization of NPs. We chose a low-dose (0.16 μg/ml) and a high-dose (25.0 μg/ml) for further experiments. We used TEM complemented with NanoSIMS50 and we could confirm that TiO_2_-NPs are uptake into cells after 1 h and 24 h treatment (Figs. 4, 5). The cells without treatment of NPs have healthy morphology and with typical 3–4 nucleoli (located inside the nucleus) (Fig. 4a, b). TEM images of treated cells reveal active intracellular transport as seen by filopodia present (cell membrane invaginations) engulfing NPs agglomerates (Fig. 4e). After 1 h and independent of the dose, NPs were already observed at the vicinity of cells and engulfed by cells (Fig. 4c, e) while at 24 h, NPs agglomerates were dispersed in the cytoplasm and localized around the perinuclear region. NPs agglomerates appeared enclosed by double-membrane cytoplasmic compartments resembling autophagosomes (Fig. 4d, f).

To improve the resolution of the characterization of the TiO_2_-NPs uptake, we combined TEM observations with NanoSIMS analysis (Fig. 5) [25]. NanoSIMS analysis confirmed that the aggregates/agglomerates were composed of TiO_2_-NPs and revealed their localization (Fig. 5). We found presence of TiO_2_-NPs in the cytoplasm, while no traces of titanium were detected in the nucleus. The NPs accumulated on the nuclear membrane without diffusion into the nucleus. The micrometric size of the titanium signals as observed in NanoSIMS50 figures indicated that the NPs were agglomerated (Fig. 5a, b).

### TiO_2_-NPs trigger an autophagic response by increasing LC3 translocation

**Fig. 6:**
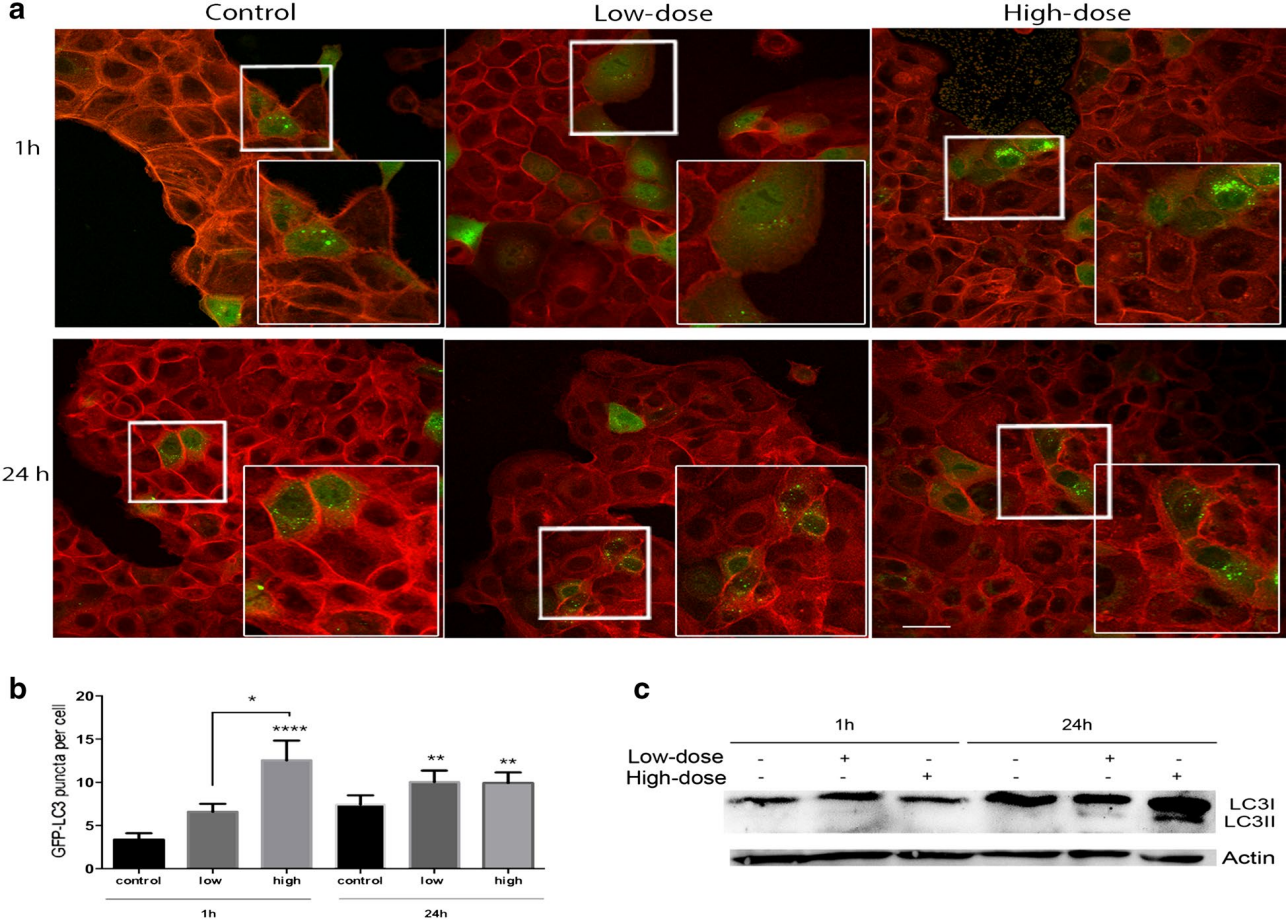
TiO_2_-NPs trigger autophagic response in HaCaT cells by forming autophagosomes and increasing LC3 translocation. Representative images of GFP-LC3 puncta. **a** Evaluation of LC3 puncta on transiently transfected eGFP-LC3-HaCaT cells treated with TiO_2_-NPs by confocal microscopy. The *green color* represents the GFP-LC3 puncta and the *red color* represents the cytoskeleton. Control cells were transfected with eGFP-LC3 plasmid and grown only in DMEM complete cell media and later cells were treated with 0.16 µg/mL (low-dose) and 25.0 µg/mL (high-dose) TiO_2_-NPs for 1 and 24 h before fixation. **b** Quantification of GFP-LC3 dots per cell (*green dots*). Up to twenty cells were analyzed. **c** Representative western blots probed with anti-LC3B antibody (Novus) from cells treated for 1 and 24 h; cells without treatment were used as a control. Data obtained was analyzed for the conversation of LC3I (cytoplasmatic) to LC3II (membrane-bound) by western blotting using an LC3B antibody. Actin was used as a loading control. Values represent mean ± SD. *P < 0.05, **P < 0.01 and ****P < 0.0001. *Scale bar* (*bottom-left*) represents 20 µm

Treated cells exhibited a distinctive mark of autophagy, autophagosomes formation (Fig. 6). We monitored LC3 protein conversion by using HaCaT cells transiently transfected with an eGFP-LC3 expressing plasmid. GFP-LC3 punctates were assessed at 1 and 24 h in eGFP-LC3 expressing cells incubated with low- and high-dose of TiO_2_-NPs and the relative number of fluorescent puncta formed per cell was quantified (Fig. 6a, b). The cytoplasmic LC3 (LC3I) inactive appears diffused throughout the cytoplasm, while activated LC3 (LC3II) appear as bright punctates (Fig. 6a). Quantification of eGFP-LC3 dots showed a significant increase after 1 and 24 h exposure to high-dose of NPs compared with untreated cells. There were equivalent numbers of puncta per cell for high- and low-doses at 24 h. These results suggest that autophagy was induced in HaCaT cells after TiO_2_-NPs treatment. We further investigated whether the cytoskeleton could be compromised during autophagic response. Treated cells were stained with red fluorescent phalloidin. For both doses of TiO_2_-NPs, the filamentous actin (F-actin) in TiO_2_NPs-treated HaCaT cells was well organized in thick bundles in the cellular cytoplasm much like with the control group (Fig. 6a). The data showed that TiO_2_-NPs do not disrupt the cytoskeleton organization.

We next detected levels of lipidated LC3II by Western blot upon 1 and 24 h for both doses of TiO_2_-NPs (Fig. 6c). The 24 h treated cells showed increased of LC3II in a dose-dependent manner.

### TiO_2_-NPs induce dysfunction of p62/NBR1 degradation

**Fig. 7:**
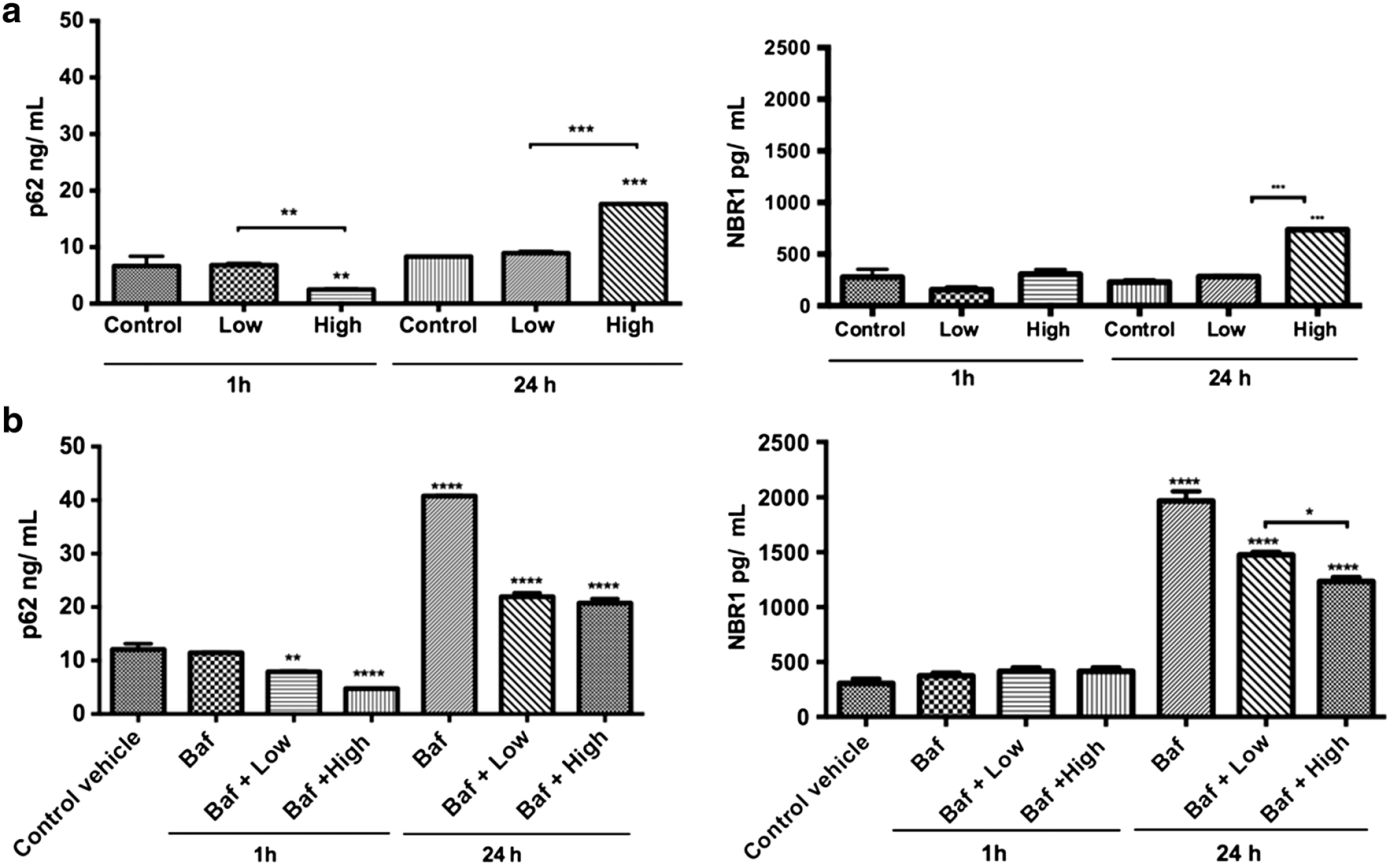
TiO_2_-NPs induce dysfunction of p62/NBR1 degradation. The p62 and NBR1 protein levels were monitored in cells treated using a colorimetric immunoassay. Cells were harvested at 1 and 24 h post-treatment and lysed in RIPA cell lysis buffer 2 containing protease inhibitors and DNase. Cell lysates were clarified by centrifugation and analyzed in p62/NBR1 assay. **a** Cells were treated to 0.16 (low-dose) and 25.0 µg/mL (high-dose) of TiO_2_-NPs for 1 and 24 h. **b** Cells co-treated with NPs and bafilomycin A1 (100 nM), a known blocker of autophagosome-lysosome fusion, and incubate in the earlier conditions. Values represent mean ± SD (n = 3). *P < 0.05, **P < 0.01, ***P < 0.001 and ****P < 0.0001

The LC3 II lipidation results showed increasing number of autophagosomes, but this is not necessarily associated to an increase in autophagy [26]. Therefore, we analysed the p62 and NBR1 protein levels (Fig. 7), selective substrates of autophagy, since activation of the autophagy leads to p62 and NBR1 degradation, and vice versa [13, 27]. Significantly higher p62 and NBR1 protein levels were detected in cells with the high-dose of NPs by time dependence. However, we observed lower levels of p62 in cells at high-doses early after treatment that could be attributed to initial autophagy induction and then blockage after accumulation of NPs.

As true autophagic function can be measured by changes in LC3-II levels in the presence of versus absence of lysosome inhibitors, we investigated the p62 and NBR1 degradation in cells co-treated with bafilomycin (Fig. 7b). Both p62 and NBR1 accumulated over time after cells were co-treated.

### Not all components of the autophagosome formation are induced in LC3 translocation

**Fig. 8:**
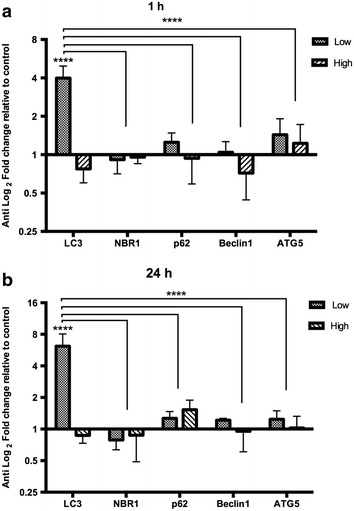
LC3 upregulation after TiO_2_-NPs treatment. Expression analysis of transcripts associated with autophagy using quantitative real time polymerase chain reaction (RT-qPCR). Data are represented as fold-changes relative to control in anti-log scale for the main autophagic markers (ATG5, Beclin1, LC3, NBR1and p62) from cells treated with 0.16 (low-dose) and 25.0 µg/mL (high-dose) of TiO_2_-NPs for 1 and 24 h. Data analysis was determined using the method 2^−ΔΔCt^, where ΔCt (treated sample) − ΔCt (untreated), and ΔCt = Ct (target gene) − Ct (reference gene). HRPT was used as the reference gene. Fold changes greater than twofold were considered biological relevant (***P < 0.001). Values represent mean ± SD (n = 3)

To better understand the autophagic response observed in cells treated with TiO_2_-NPs, we analyzed the expression of autophagy markers (LC3, NBR1, p62, Beclin 1 and ATG5) by RT-qPCR (Fig. 8). Beclin 1 and ATG5 are genes involved in the initiation of autophagosomes formation [26].

The LC3 II expression was significantly higher in cells after 1 and 24 h treatment with low-dose of TiO_2_-NPs (four and sixfold upregulation) (Fig. 8a, b). However, we did not observe significant differences in gene expression relative to cells that had been treated with a high dose of NPs. The data demonstrate a time-dependent upregulation of LC3 mRNA levels in low-dose of NPs treated cells with NPs corroborating with an increase of eGPF-LC3 puncta number and LC3I/LC3-II conversion levels described above. All these observations are consistent with autophagy induction. However, for high-dose treatment, we could not find an increase in LC3 gene expression.

## Discussion

TiO_2_-NPs applied to this study display aggregation in cell media as previously described despite ultrasonication [28]. The difference in particle sizes between powder and solutions is expected since the molecules present in the cell culture media/physiological solutions will surround NPs, forming the so-called protein corona. In the case of NPs in cell media, the NPs protein corona is composed by serum proteins and essential amino acids that would modify their hydrodynamic size [29]. In this study, we selected 18 nm anastase TiO_2_-NPs for our experiments as this size to be suitable for efficient cellular uptake and commonly used in consumer-based products [16].

To date, a large number of in vitro studies have shown that TiO_2_-NPs can induce autophagy in endothelial cells, glioblastoma and primary keratinocytes. However, these autophagy inductions have been mostly linked to cytotoxicity or ROS generation events [4–6]. We have shown that TiO_2_-NPs elicit an autophagic effect in a dose-dependent manner in the absence of cytotoxicity.

On the one hand, the presence of GFP–LC3 puncta dots and autophagosomes in both doses is interpreted as a sign of autophagy induction, however it may be in fact an impairment of autophagy. On the other hand, we could not find an increase in LC3 gene expression that justifies the higher number of observed GFP-LC3 puncta and the higher levels of LC3 protein, at least for high-dose treatment. The level of a protein at a certain time is the result of combined actions between synthesis and degradation machinery. Since LC3 protein synthesis seems to be downregulated at high-dose treatment, the higher levels of LC3 protein observed is probably due to the impairment of LC3 degradation; therefore, autophagy is also impaired. Furthermore, ATG5, Beclin1, NBR1 and p62 genes, genes involved the formation of autophagosomes were not affected. The correlation between protein and gene expression levels is not always straightforward. We hypothesize that these divergent findings can be due to post-translational modifications, specifically of the LC3 protein. For example, a rapid phosphorylation of LC3 within 15–30 min has been reported in hepatoma cells, promoting LC3 I to LC3 II conversion [26, 30].

Autophagy induction is a well described effect among inorganic NPs including TiO_2_-NPs and associated with the increase of autophagosomes [4, 9, 30–32]. Herein, the high-dose treatment appeared to slow the degradation of autophagic substrates than the low-dose, over time. This accumulation of cargos was also confirmed in co-treated cells with bafilomycin. Briefly, a high-dose of NPs may supply more autophagic cargos without an increase in the degradation of cargos; thus resulting in high accumulation of autophagic vacuoles. Recent studies with gold and silica NPs suggest that these highly stable NPs may slower the degradation of autophagic substrates proteins modulating the autophagic effect and suggesting autophagy blockage [32]. Though NBR1 degradation in cells treated has not been reported before, the accumulation of NBR1 has been reported along with p62 levels when cellular autophagy was investigated in the presence of other stressors [13, 27]. However, the accumulation of autophagosomes and high levels of LC3II protein is not sufficient to conclude that the exposure to TiO_2_-NPs can activate autophagy.

Our findings suggest that cells may degrade autophagic substrates slowly due to the existence of metal oxide NPs, which are difficult to digest in high-dose and over time. This largely uptake of aggregated NPs at high-dose may overload the autophagy capacity. It was recently shown that fullerene NPs can impair autophagy flux by an overload of NPs and consequently interrupt autophagosome-lysosome fusion [6]. We cannot clarify why at low-dose the degradation is not compromised. Recently, iron oxide, gold and silica NPs exhibited a dispersity-dependent autophagic effect in cells under non-cytotoxic conditions, which supports our findings [33].

Autophagy has an important role on cellular functions, either promoting cell survival under stress conditions, cell death or organelle quality control. Nevertheless, the key function of autophagy is degradation. From the perspective of degradation, autophagy induction and autophagy impair have distinct effects; the first enhances degradation, while the latter prevents it [34]. Thus, autophagy induction and autophagy impair have different biological effects. Autophagy dysfunction is then an initial event that has been implicated in various human pathologies including cancer, neurodegeneration, abnormal immune responses and premature ageing. And so, autophagy regulation has become a novel therapeutic strategy [12].

Although more in-depth studies are needed to clarify the mechanism, our data suggests a potential way for modulate autophagy by tuning their dose. Various NPs have been considered as potential therapeutic autophagy modulators. Liposomes and hard NPs such as gold NPs and quantum dots have been reported as autophagy activators with anticancer effect. Alternatively, other NPs (e.g., graphene oxides and fullerenes) have shown to block autophagic flux [35, 36]. However, the complexity of signalling means modulating autophagy with NPs could bring safety concerns. Earlier studies of NPs’ impact on cell systems suggest that dosage is a critical parameter in pathway modulation [37, 38]. This type of study could deliver additional insights in enabling better safe-by-design strategies for NPs used in consumer products and/or in drug delivery therapy.

## Conclusions

The findings presented here provide evidence of the autophagic effects by TiO_2_-NPs in a dose-dependent manner and under non-cytotoxic conditions. Depending on the initial dose, TiO_2_-NPs can switch the autophagic response of keratinocytes between blockage and induction. TiO_2_-NPs-mediated autophagy induction at low-dose can be interpreted as a standard mechanism by which cells attempt to increase the clearance of NPs. While the halt in autophagy at high-dose may indicate that the cellular response cannot cope with a NPs overload and thus show a deficient degradative capacity. The lack of cell toxicity and ROS generation, despite the presence of the dysfunctional autophagic effect suggests that in future studies, other targets of nanotoxicity should be assessed in addition to those targeted by traditional assays. In addition to clarifying the mechanism by which TiO_2_-NPs induce autophagosome accumulation, these results have increased our understanding of the impact that TiO_2_-NPs has on keratinocytes and enhanced our knowledge about particle-cell interactions.

## Methods

### NPs preparation and characterization

TiO_2_-NPs with 18 nm core diameter (as provided by manufacturer) were obtained from NanoGrade, Nanocomposix (San Diego, CA) and stock solutions were prepared in complete cell medium and dispersed by sonication according the previously described protocol used by [39]. NPs were characterized using zeta potential (Z-potential) and dynamic light scattering (DLS) analysis. The measurements were assessed with a Malvern Zetasizer Nano series V5.03 (PSS0012-16 Malvern Instruments, Worcestershire, UK) and the analysis program dispersion technology software (Malvern Instruments, UK). The polydispersity index (PDI) was also assessed. Measurements were conducted in triplicates. Images of TiO_2_-NPs in cell medium were examined in a Jeol transmission electron microscopy 1230 (TEM) (Japan) at 100 kV.

### Cell line and treatment

Human keratinocyte cells (HaCaT) were purchased from the CLS Cell Lines Service (Eppelheim, Germany) [17, 40]. HaCaT cells were cultured in Dulbecco’s modified Eagle’s medium, high glucose (DMEM) (Life Science Technologies Europe BV, Sweden) supplemented with 10 % fetal bovine serum (FBS), penicillin (100 U/ml), streptomycin (100 mg/ml) and glutamine (2 mM) (all from Life Science Technologies Europe BV, Sweden), and maintained in a humidified atmosphere at 37 °C with 5 % CO_2_. The cells were sub-cultured every 4–6 days or when they reached 80–90 % confluency. For treatment, 8.0 × 10^4^–1.0 × 10^5^ cells/ml were used and seeded in 96- and 6-well microplates or T-75 flasks depending of the assay. Before 1 and 24 h-treatments with NPs, cells were allowed to recover, attach and proliferate for 24 h. For each treatment and assay, new stock solutions of NPs in complete DMEM were prepared to provide concentrations of 0.16 and 25.0 μg/ml of TiO_2_-NPs (except for the cytotoxicity assays), then sonicated 30 min to disperse NPs and vortexed 1 min.

### Cell viability

The 3-(4, 5-dimethylthiazol-2-yl)-2, 5-diphenyl tetrazolium bromide (MTT) and neutral red (NR) assays were used to assess cell viability [23, 41]. The cells were seeded at 8.0 × 10^4^ cells/ml in 96-well plate as described above. For the MTT assay reagent stock was prepared by dissolving MTT (Sigma, USA) in phosphate buffered saline (PBS) at 5 mg/ml and 0.16 μm filtered. The stock solution was then added to the cell medium in a 1:10 (v/v) volume ratio and the cells were re-incubated for 2–4 h. The cells were subsequently washed and the dye was extracted with 100 % dimethyl sulfoxide (DMSO) solution. After 10 min of mild shaking, the absorbance of formazan product formed was measured at λ = 570 and 640 nm (background) by a microplate reader (BioTeK, USA). The cell viability was expressed as the percentage of cells absorbance treated and subtracted with background absorbance in comparison with control cells (untreated cells).

For NR assay, cells were also seeded in 96-well culture plates and treated in the appropriate period. The plates were then incubated for 2 h with a cell culture medium containing neutral red at 40 µg/ml as described elsewhere [23]. The cells were subsequently washed, the dye was extracted in each well and the absorbance was read at 540 nm using a microplate reader (BioTeK, USA). NR assay was also used to assess lysosomal activity. For MTT and NR assay, series of concentrations ranging from 0.0 to 25.0 μg/ml were employed.

### Detection of oxidative stress

Intracellular reactive oxygen species (ROS levels) were measured using the dichlorodihydrofluorescein diacetate (DCFH-DA) assay. DCFH-DA is a lipophilic cell permeable compound that is deacetylated in the cytoplasm to DCF by cellular esterases. Upon oxidation by radicals such as hydroxyl, peroxyl, alkoxyl, nitrate and carbonate, DCFH-DA turns to highly fluorescent 2′, 7-dichlorofluorescein DCF [42]. Briefly, HaCaT cells were seeded in black 96-well plates with a transparent bottom and incubated with TiO_2_-NPs (0.0–25.0 μg/mL) for 1 and 24 h. Cells were then washed with PBS and incubated with 5 μM DCFH-DA in PBS for 30 min at 37 °C. Thereafter, cells were washed with PBS and fluorescence was recorded with excitation at 485 nm and emission at 530 nm) using a plate reader (BioTek, Synergy, USA) with measurements done in triplicate. Hydrogen proxide (H_2_O_2_, 100 μM) was used as a positive control. The results are presented as mean ± standard deviation (SD). Values are expressed as relative fluorescence units (RFU) normalized.

### Electron microscopy

The cells exposed to TiO_2_-NPs were fixed in 2 % glutaraldehyde in 0.1 M sodium cacodylate and 0.1 M sucrose, pH 7.4 and post-fixed with 1 % OsO4 in 0.15 M sodium cacodylate buffer. Cells were dehydrated and immersed in a mixture of absolute ethanol and Epon 812 (1:1) and then embedded in pure Epon 812. BEEM^®^ capsules containing polymerized Epon were placed upside down at a straight angle to the cell layer and were polymerized for 48 h at 60 °C. The Epon layer with the BEEM^®^ capsules was removed from the Petri dish, leaving the cells on the surface of the Epon layer. The capsules were then removed with tweezers and ultrathin sections (60 nm) were cut with a diamond knife (Diatome, Switzerland) on a Reichert-Jung ultracut (Austria) and collected on Formvar-coated Cu 100-mesh grids. The sections were counterstained with uranyl acetate and lead citrate and examined in a Jeol 1230 TEM (Japan) at 100 kV.

### Confocal microscopy

To measure GFP-LC3 puncta formation in cells exposed, HaCaT cells were allowed to adhere to glass coverslips on 35 mm dishes at a density of 8 × 10^4^ cells/mL in DMEM (Invitrogen, USA) supplemented with 10 % FBS and incubated for 24 h. The medium was then replaced with Krebs–Ringers glucose buffer (KRG) complete, and each sample transfected with pEGFP-LC3, Plasmid 24920 (Addgene, USA) using TurboFect (Thermo Scientific, USA). After 10–12 h-transfection, the medium was removed and cells treated as described previously. Cells were then fixed in 3 % paraformaldehyde (PFA) for 15 min, washed three times in PBS and permeabilized with Triton X-100 for 45 s. The cells were labelled with phalloidin 568 to visualize actin filaments. The CLSM imaging was conducted using a LSM 700 (Carl Zeiss, Germany) equipped with a C-Apochromat 63 objective (Carl Zeiss, Germany). The intracellular distribution of LC3 puncta was quantitatively evaluated using ImageJ (1.480 version). The results are expressed as mean ± SD of puncta per cell obtained from up to 25 cells.

### Secondary ion mass spectrometer analysis (NanoSIMS)

The secondary ion mass spectrometer (SIMS) acquisitions were performed on a NanoSIMS50 instrument (CAMECA, France) in imaging mode. The primary ion bombardment of caesium was accelerated at 8 kV with the primary current was 1.5 pA. These conditions allowed estimating the probe size to 100 nm [43, 44]. The samples were scanned with a matrix of 256 × 256 pixels and a pulverization time of 30 ms per pixel (~33 min per image). The emitted secondary negative ions from the nano volume were selected in mass with an instrument tuned for a mass resolution power ∆M/M of 4500. The mass recorded and counted simultaneous were the ^12^C^14^N-cluster (m = 26.00307 uma), the ^31^P-ion (m = 30.97376 uma) and the ^34^S-ion (m = 33.96786 uma). Titanium was detected as cluster TiO. The two majors isotopes, ^46^Ti (8.25 %) and ^48^Ti (73.8 %), were detected as ^16^O^46^Ti-cluster (m = 61.94854 uma) and 16O48Ti- cluster (m = 63.94286 uma) to check the isotopic ratio and then to ensure that no mass interference induced an analysis artefact. For example, to solve the interfering with ^32^S^16^O2- (m = 63.96190 uma) a mass resolution power of 3360 was required. The data was treated using ImageJ (1.45 version) [45] and the NRIMS ImageJ Analysis module [46]. The image of CN-cluster, corresponding mainly to the nitrogen signal from proteins and melanin, provide an image with a contrast similar to an optical microscopy image, which allows distinguish without ambiguity the cell, the cytoplasm and the nucleus. Phosphorus mostly comes from the DNA while sulfur ions are essentially representing di-sulfid bridges present in proteins [46]. The CN- image scale goes from black to white with increasing intensity, while the titanium is represented uniquely in red. Both images, CN and TiO, were overlapped to localize precisely the titanium in the subcellular compartments.

### NBR1 and p62 degradation assays

For the quantitative determination of human NBR1 and p62 autophagic cargos, colorimetric and immunoassay kits, NBR1 (ADI-900-211), and p62 (ADI-900-212) ELISA from Enzo Life Sciences, Inc. (USA), were employed. All procedures were performed according to manufacturer’s protocol and in triplicate.

### Immunoblotting

Cells extracts were prepared in RIPA lysis buffer (Thermo Scientific Pierce, USA) with a protease inhibitor mixture (Roche Molecular Biochemicals, USA) at 4 °C. Cell lysates were collected and analyzed for protein content using the BCA protein assay kit (Thermo Scientific Pierce, USA). The lysates were separated by 12 % SDS–polyacrylamide gels and transferred onto nitrocellulose membranes (Amersham Pharmacia Biotech, USA). Immunoblot analyses were performed using mouse polyclonal antibody against LC3 (NB100-2220, Novus Biologicals, USA) and rabbit polyclonal antibody against actin (PA1-16889, Thermo Scientific Pierce, USA). Actin was used as a loading control. The secondary antibodies used were goat anti-mouse and anti-rabbit Poly-HRP Antibodies (Thermo Scientific Pierce, USA). Proteins were visualized using ECL detection kit (Amersham Pharmacia Biotech, USA). At least two duplicates were done.

### Quantitative reverse transcription PCR (RT-qPCR)

Gene expression level changes of key genes involved in autophagy were analyzed by RT-qPCR in HaCaT cells at 25.0 and 0.16 μg/ml of TiO_2_-NPs. Cells were seeded in 6-well plates in complete DMEM media for 24 h as previously described. Complete DMEM medium consists of DMEM supplemented with 1 % penicillin–streptomycin and 10 % fetal bovine serum (Invitrogen Life Science Technologies, USA). TiO_2_-NPs prepared in DMEM medium complete were added into cells with a final concentration of 0.16 and 25.0 μg/ml (low and high dose, respectively) for 1 and 24 h-treatment. DMEM medium complete only was used as the untreated control. Total RNA was then extracted from TiO_2_-NPs-exposed cells using PureLink^®^ RNA Mini Kit with Trizol (Invitrogen Life Science Technologies, USA) and reverse transcribed to cDNA by Maxima First Strand cDNA Synthesis Kit (Thermo Scientific, USA). Prior DNase treatment was performed with DNase I, Amplification Grade (Invitrogen Life Science Technologies, USA). RNA yield was determined using a NanoDrop spectrophotometer (Thermo Scientific, USA). RNA integrity number (RIN) was also assessed using Agilent 2100 Bioanalyzer with Agilent RNA 6000 Nano (Agilent Technologies, USA). Only RIN-values above eight were considered. All procedures were performed according to manufacturer’s protocol. RT-qPCR was performed using SYBR^®^ Green Master Mix Real-Time PCR Master Mix (Invitrogen Life Science Technologies, US) on Applied Biosystems 7500 Fast Real-Time PCR System (Invitrogen Life Science Technologies, USA). Primer sequences are listed in (Table 2) and were reported previously [47]. HPRT, hypoxanthine phosphoribosyl transferase, was used as reference gene. Only NBR1 primers were designed using Primer3 software http://Frodo.wi.mit.edu/primer3. Changes in gene expression were normalized based on average threshold cycles (Ct) values of reference gene HRTP for each trial. Data analysis was determined using the method 2^−ΔΔCt^, where −ΔΔCt = ΔCt (treated sample) − ΔCt (untreated), and ΔCt = Ct (target gene) − Ct (reference gene) [48]. All samples including no-RT (reverse transcriptase) and no-template controls were analyzed in biological triplicates and technical duplicates.

**Table 2:** Primers used in RT-qPCR

Primer name	Sequence (5′–3′)
Atg5 forward	GCAGATGGACAGTTGCACACA
Atg5 reverse	TTTCCCCATCTTCAGGATCAA
Beclin1 F	CTGGACACGAGTTTCAAGATCCT
Beclin1 R	TGTGGTAAGTAATGGAGCTGTGAGTT
hLC3B F	ACCATGCCGTCGGAGAAG
hLC3B R	ATCGTTCTATTATCACCGGGATTTT
p62 F	AGGCGCACTACCGCGAT
p62 R	CGTCACTGGAAAAGGCAACC
NBR1 F	TGGGCAAATGAGTGTGTGTG
NBR1 R	TTGCCCCTGTCCAAGTTTTG
HPRT F	TGACACTGGCAAA ACAATGCA
HPRT R	GGTCCTTTTCACCAGCAAGCT

### Statistical analysis

One- or two-way analysis of variance (ANOVA) where appropriate, followed by Tukey test for post hoc comparisons was performed using GraphPad Prism version 6.05, GraphPad Software (La Jolla California USA, http://www.graphpad.com). Results were considered statistically significant if the *P* value was less than 0.05.

## Abbreviations

ANOVA: analysis of variance
Ct: threshold cycle
DCFH-DA: dichlorodihydrofluorescein diacetate
DLS: dynamic scattering light
DMEM: Dulbecco’s Modified Eagle Medium
eGFP: green fluorescent protein
FBS: fetal bovine serum
HPRT: hypoxanthine phosphoribosyl transferase
KRG: Krebs–Ringers glucose buffer
LC3: microtubule-associated protein 1A/1B-light chain 3
MTT: 3-(4, 5-dimethylthiazol-2-yl)-2, 5-diphenyl tetrazolium bromide
NanoSIMS: secondary ion mass spectrometer
NPs: nanoparticles
NR: neutral red
PBS: phosphate buffered saline
PDI: polydispersity index
PFA: paraformaldehyde
RFU: relative fluorescence units
RIN: RNA integrity number
RIPA: radioimmunoprecipitation assay buffer
RNA: ribonucleic acid
ROS: reactive oxygen species
RT: reverse transcriptase
RT-qPCR: quantitative reverse transcription PCR
SDS-PAGE: polyacrylamide gel electrophoresis
TEM: transmission electron microscopy
